# Suitable classification of mortars from ancient Roman and Renaissance frescoes using thermal analysis and chemometrics

**DOI:** 10.1186/s13065-015-0098-z

**Published:** 2015-04-24

**Authors:** Mauro Tomassetti, Federico Marini, Luigi Campanella, Matteo Positano, Francesco Marinucci

**Affiliations:** Department of Chemistry, University of Rome, “La Sapienza” P.le A. Moro 5, I-00185 Rome, Italy

**Keywords:** Mortars, Ancient Roman frescoes, Renaissance frescoes, Thermal analysis, Principal component analysis (PCA)

## Abstract

**Background:**

Literature on mortars has mainly focused on the identification and characterization of their components in order to assign them to a specific historical period, after accurate classification. For this purpose, different analytical techniques have been proposed. Aim of the present study was to verify whether the combination of thermal analysis and chemometric methods could be used to obtain a fast but correct classification of ancient mortar samples of different ages (Roman era and Renaissance).

**Results:**

Ancient Roman frescoes from Museo Nazionale Romano (Terme di Diocleziano, Rome, Italy) and Renaissance frescoes from Sistine Chapel and Old Vatican Rooms (Vatican City) were analyzed by thermogravimetry (TG) and differential thermal analysis (DTA). Principal Component analysis (PCA) on the main thermal data evidenced the presence of two clusters, ascribable to the two different ages. Inspection of the loadings allowed to interpret the observed differences in terms of the experimental variables.

**Conclusions:**

PCA allowed differentiating the two kinds of mortars (Roman and Renaissance frescoes), and evidenced how the ancient Roman samples are richer in binder (calcium carbonate) and contain less filler (aggregate) than the Renaissance ones. It was also demonstrated how the coupling of thermoanalytical techniques and chemometric processing proves to be particularly advantageous when a rapid and correct differentiation and classification of cultural heritage samples of various kinds or ages has to be carried out.

**Graphical abstract:**
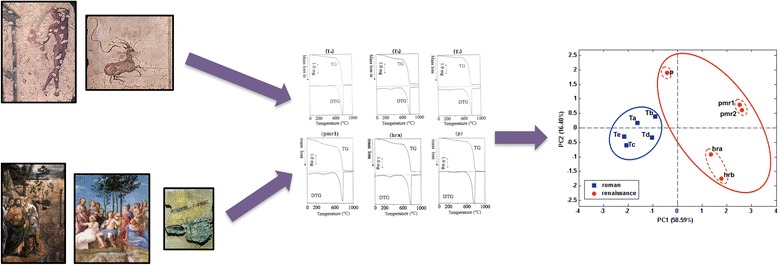
PCA analysis of TG data allows differentiating mortar samples from different ages (Roman era and Renaissance).

## Background

Mortars consist of an intimate mixture of sand or pozzolan, binder and water [1]. Studies on ancient mortars are relatively recent [2-4] and have mainly focused on the identification and characterization of their components in order to assign them to a specific historical period, after accurate classification [5]. More recently, numerous authors have focused specifically on the characterization of mortars. For instance, Duran *et al.* [3] have used different experimental techniques (TG, DTA, XRD, FTIR, SEM-EDX, Bernard calcimeter, granulometry, mercury intrusion porosimetry and mechanical strength tests) to characterize mortars taken from the walls of three different buildings of Seville (Spain). On the other hand, Pavia and Caro [6], on the basis of a detailed petrographic analysis, postulated that the ancient Romans used a pure carbonate rock for lime making. Consequently, Roman mortars were probably made using a non-hydraulic, or feebly hydraulic, lime and their hydraulicity was mainly due to the addition of pozzolans. These claims are in good agreement with the findings of Degryse *et al.* [7] who used optical microscopy and XRD analysis to study ancient Roman mortars discovered in southwestern Turkey. They suggested that the best type of mortar to be used for further on-site conservation and restoration consists of a mixture of lime and crushed volcanic sediments or rock, that is, of materials very similar to the original materials used also in many other ancient Roman sites. On the other hand, a particular, but more interesting paper was published by E. Pecchioni *et al.* [8] reviewing some examples of ancient mortars used in the Florence area. These authors emphasized how mortars play a central role as supports for frescoes, mural paintings and as the white rendering of Renaissance architecture. Their mineralogical and petrographic study of the mortar’s aggregate revealed how, in this case, sand was used as an aggregate rather than crushed stone. Further, this type of investigation provided information on the stone used to make the lime, as well as on the technology used in the manufacturing phases and in kiln firing, and enabled the binder/aggregate ratio to be evaluated. Other recent studies have focused on the processes of deterioration of mortars and the influence of these alterations on the adjacent materials. In this connection J. Elsen [9] published a review on microscopic studies of historic mortars. The review was accompanied by an extensive bibliography and postulated that the first step in mortar characterization schemes is optical microscopy to identify aggregates and the various mineral additions, binder type, the binder-related particles, the pore structures and how this technique acts as a valuable aid in the damage diagnosis of degraded historic mortars. Lastly, several instrumental analytical methods were used in recent research to study the composition of ancient Roman mortars and stuccos [10,11], above all for the purpose of identifying any differences in their composition in order to distinguish and accurately classify these two different types of artifacts [12]. On the other hand, Conti *et al.* [4] have used different analytical techniques (TG-DSC, XRD, FTIR and SEM-EDX) to characterize mortars from different structures of the archaeological site of Baradello (Italy), in order to define its building chronology.

Over the past few years we have studied and characterized several mortar samples from ancient Roman (2^nd^ Century AD) and Renaissance (16th Century) frescoes [13,14]. On the other hand, recently we showed how thermal analysis, in particular thermogravimetry (TG-DTG), coupled with chemometric methods, can be a valid tool for the characterization and classification of several types of archaeological finds and cultural heritage, e.g. pigments [15], marbles [16], pottery [17], fossil bones [18], and so on. Therefore, we decided to apply thermogravimetric analysis also to several mortars sampled from different ancient Roman and Renaissance frescoes to determine whether, also in the case of ancient mortars, the simple combination of thermal analysis and chemometric methods could be used to obtain a fast but correct classification of ancient mortar samples of different ages.

### Historical information

#### Samples from ancient Roman frescoes (2nd century A.D.)

Samples were taken from the “Stazione Termini” frescoes, which are part of the “Piazza dei Cinquecento Complex”.

**Figure 1:**
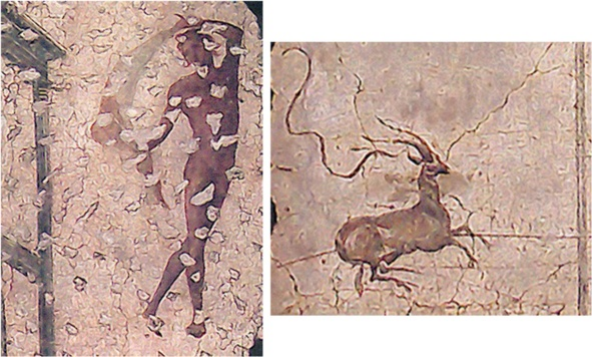
Details of two of the studied Ancient “Termini Station” Roman frescoes from Museo Nazionale Romano, Rome, Italy.

As early as 1862, on the occasion of the work to construct the Rome Central Railway Station commissioned by the Holy See in the area previously occupied by Villa Montalto, several imperial age rooms came to light. During work to extend Central Station and to construct Line B of the underground in 1947, much of the entire quarter was excavated, uncovering also the finds made a century earlier. The building unearthed by the excavations belonged to a building complex constructed during the 2^nd^ century A.D. between the patrician quarter of the Viminale and the city walls. Outstanding in this complex was a building, which was also the best documented, comprising a “Domus” and the “Balnea”. It included the remains of wall paintings and mosaics, which were removed at the time of the work on the Central Station extensions and are now conserved in the Museo Nazionale Romano. The restoration work yielded very small samples, which were obtained from the frescoes and have been examined in the present work (see Figure 1). The presence of a statue of Faustina Maggiore and of the fistula of Vibia Aurelia seem to indicate that early owner was indeed Faustina Maggiore, before she became empress as the bride of Antoninus Pius. The Domus would later have been inherited by her daughter Faustina Minore, and then by the granddaughter Vibia Aurelia Sabina.

#### Specimens taken from Renaissance frescoes

**Figure 2:**
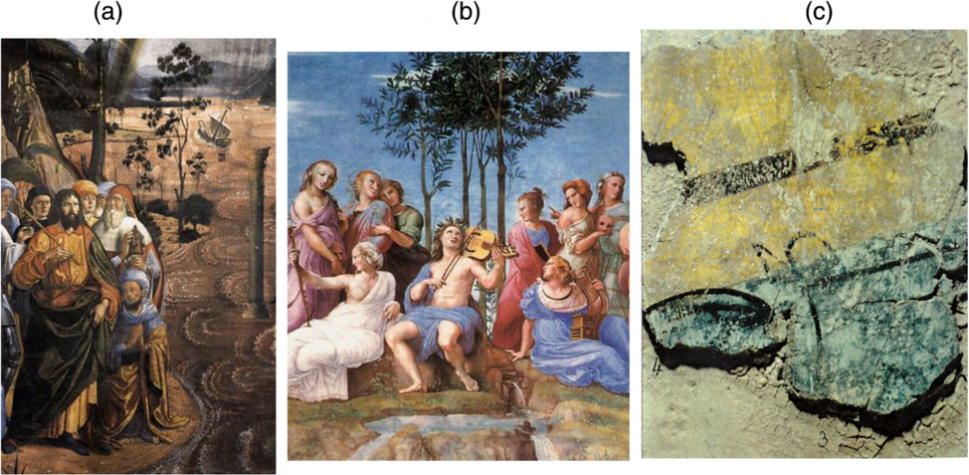
Details of frescoes from the Sistine Chapel or ancient Vatican Rooms (Vatican City). **(a)** C. Rosselli, “Il Passaggio del Mar Rosso”; **(b)** Raffaello, “Il Parnaso”; **(c)** fragment from the “Room of Heliodorus” glued on pottery support.

“Passaggio del Mar Rosso”: from 1481 to 1483 Cosimo Rosselli took part in the decoration of the Sistine Chapel, the fresco of which contained different scenes such as “Il sermone della Montagna” and “l’Ultima Cena”, “Mosè riceve le tavolo della legge”, and lastly “Il passaggio del Mar Rosso” (Figure 2a). According to the commissioner’s desire it was necessary to present the sacred events in their ideological significance. This was fully grasped by Rosselli who performed these works not only painstakingly but also gave them an austere and highly dignified tone [19]. Also this fresco underwent a restoration in relatively recent times during which the stucco was consolidated, and clearing and reintegration of the picture carried out.

“Il Parnaso”: At the end of 1508 Raffaello was invited to Rome to help in the decoration of the rooms of the new apartments of Julius II and he began the main undertaking of his relatively short life starting in the middle room known as “della Segnatura” [20]. On the wall facing onto the Belvedere courtyard, Raffaello depicted the scene that glorifies poetry, namely “Il Parnaso” (Figure 2b), despite the fact that the irregular surface of the wall must have made it very difficult to achieve a smooth pictorial composition. Raffaello used groups of small trees placed in the background and divided the wall into four parts, arranging the characters into groups linked together by secondary figures, synchronizing the whole with the curve of the arch, which descends beyond the top of the window [14]. During the restoration of the fresco carried out in 1962 it was found that Raffaello, in order to gain extra space, lowered the 15th century window opening, which was originally much higher.

“Stanza di Eliodoro”: the specimen examined consisted of two tiny fragments (glued on pottery support, see Figure 2c) of a fresco located originally in the Room of Heliodorus (Ancient Vatican Rooms), specifically in the zone of the “chiaroscuri”, that is the outer area framing the four large frescoes located in that room.

## Results and discussion

**Figure 3:**
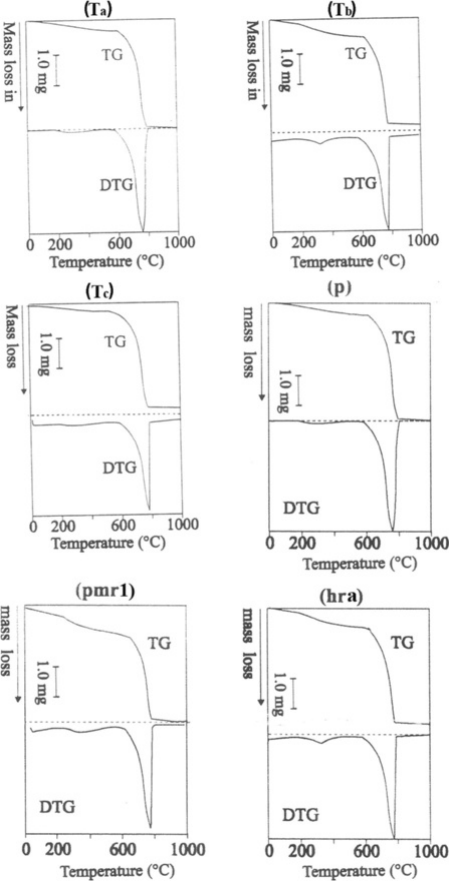
Typical TG and DTG curves of the “Termini Station” samples (Ta-Tc) and of samples from the Vatican Rooms (p, pmr1, and hra); heating rate 10°C min^−1^ under an air stream of 100 cm^3^ min^−1^.

Typical TG and DTG curves for the various samples are shown in Figure 3.

**Figure 4:**
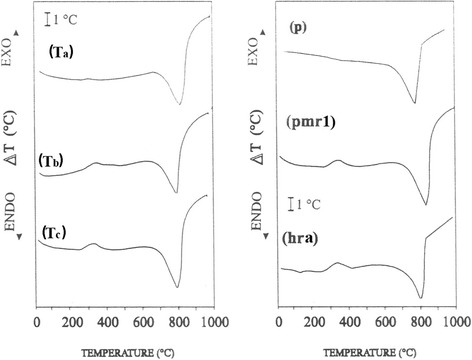
Typical DTA curves measured on the “Termini” samples (Ta-Tc) and on samples from the Vatican Rooms (p, pmr, and hra); heating rate 10°C min^−1^ under an air stream of 100 cm^3^ min^−1^.

Examination of the thermograms reveals above all a large step, the DTG peak of which occurs at around 750°C (see Table 1), corresponding to the process of decomposition of the carbonates contained in the binder (calcium carbonate). A further two smaller steps are visible at lower temperatures, the first (around 100°C) due to the loss of the small moisture content; the second, around 310–330°C, is characterized by a modest exothermic process as can be seen in several of the typical DTA curves in Figure 4.

**Table 1:** **Main thermogravimetric data**

**Sample name**	**% mass loss (a)**	**% mass loss (b)**	**DTA T**_**peak**_ **(b)**	**% mass loss (c)**	**DTA T**_**peak**_ **(c)**	**Residue at 1000°C**
pmr1	2.31	4.37	325	13.71	780	79.34
pmr2	1.98	5.62	320	13.25	775	79.73
hra	1.16	3.48	335	14.31	775	80.97
hrb	1.07	5.39	345	14.19	770	79.14
p	1.10	3.27	300	19.3	760	75.5
Ta	1.00	2.90	318	40.1	766	55.9
Tb	1.50	4.80	320	39.5	760	54.6
Tc	0.60	2.30	324	41.2	767	55.2
Td	1.50	3.80	330	37.9	759	56.4
Te	0.90	2.90	325	39.1	754	56.7

RSD% ≤ 1.2 for all T values.

RSD% ≤ 1.5 for all mass loss values and residue at 1000°C.

This is therefore probably due to the oxidative breakdown of trace quantities of organic substances. The principal data (mass loss, DTA peak temperatures, final % residues) of the main TG and DTA steps are summarized in Table 1.

Cursory examination of the data reveals that the various step temperatures do not differ appreciably in any of the samples, as is true also for the amount of moisture loss in the first step. However, a much higher mass loss in step three (and, as a consequence, a much smaller residue at 1000°C) is observed in the samples obtained from the Ancient Roman frescoes compared with those of the Renaissance frescoes. This would seem to point to a higher percentage of binder and a smaller percentage of filler in the former than in the latter.

**Figure 5:**
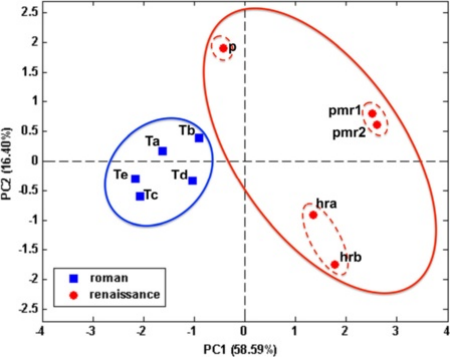
PCA on all the data reported in Table 1 after autoscaling. Scores plot.

All the data reported in Table 1 were therefore processed after autoscaling by Principal Component Analysis [21,22] for the purpose of demonstrating how thermal data alone, simply with the help of chemometric projection techniques, are sufficient to separate mortars belonging to frescoes of different ages into different clusters. Indeed, the representation of the samples onto the space spanned by the two significant principal components (see Figure 5) clearly evidences a separation between Roman and Renaissance frescoes, and also the subgrouping of the latter.

**Figure 6:**
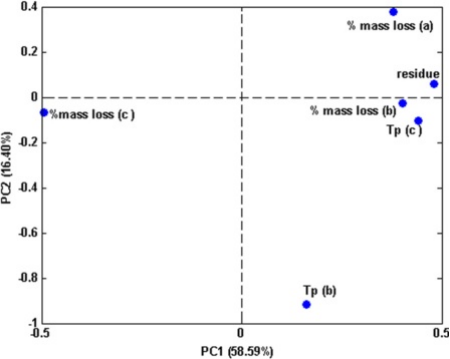
PCA on all the data reported in Table 1 after autoscaling. Loadings plot.

Indeed, results show that the two groups of samples (i.e. ancient Roman and Renaissance frescoes) are clearly separated along the first principal component, the differentiation being related in particular to the mass loss due to the carbonate decomposition step and the percentage of final TG residue at 1000°C, as can be observed in the loadings represented in Figure 6. In particular, the first principal component is mostly made of the contribution of the third identified thermogravimetric step, corresponding to the carbonate decomposition (both in terms of mass loss – with a high negative loading - and peak temperature, which has a positive value), together with the residue at 1000°C and the mass loss in the second TG step (both with positive loadings). On the other hand, the second principal component is mainly characterized by the moisture content and the peak temperature of the second TG step, the former with positive and the latter with negative loadings, respectively. This fully confirms the preliminary considerations made on the data reported in Table 1. The loading representation showed that also the mass loss of the organic traces exerts however a certain role in the separation of different samples along the first principal component. It is also interesting to note that, in the case of the Renaissance samples, three subclusters can readily be identified, which refer to the three different frescoes from which the samples were obtained. In this case, inspection of the loadings (Figure 6) confirms that the separation into three subclusters corresponding to the samples coming from the three different Renaissance frescoes, which occurs along the second PC, is essentially due to the DTG peak temperature of the organic substance loss step and to the moisture mass loss.

### Experimental

#### Samples

The ancient Roman frescoes studied are currently preserved in the Museo Nazionale Romano (Terme di Diocleziano, Rome, Italy) and denoted as “Termini Station” samples in the present paper, as the site from which they originate is near the present-day Termini Central Railway Station (Rome, Italy). They have been labeled as Ta, Tb, Tc, Td, Te (see examples in Figure 1). The Renaissance fresco samples come from the Sistine Chapel or the “Old Vatican Rooms” (Vatican Museum, Rome, Italy) and belong to three different frescoes known as: “Il Passaggio del Mar Rosso” (pmr), located in the Sistine Chapel, “Il Parnaso” (p), and lastly the specimen from the “Room of Heliodorus” (hr), located in the Old Vatican Rooms; examples are shown in Figure 2.

#### Instrumental techniques applied

10–20 mg of each sample (i.e., few granules of material coming from the *intonaco/intonachino* or *intonaco/arriccio*, in the case of Roman and Renaissance frescoes, respectively) obtained during the restoration of the frescoes were gently ground up and placed in an alumina crucible. They were then subjected to controlled thermal scanning from room temperature up to 1000°C at a heating rate of 10°C min^−1^ in an air flow of 100 cm^3^ min^−1^, using a Mettler TG 50 thermobalance connected to a Mettler TC 10 A processor and a Dupont TA 1200 DTA instrument coupled to a TA 2000 processor [5,23].

## Conclusions

In conclusion, PCA analysis has unequivocally shown how the ancient Roman mortar samples are much richer in binder (calcium carbonate) and contain less filler (see TG residue values at 1000°C), than the Renaissance mortar samples: indeed, the difference between the higher values of the TG residue of the first five samples in Table 1, which correspond to Renaissance frescoes, and the lower values of the remaining last five, which are the Roman ones, can be ascribed to a higher amount of filler in the former. This is likely due to the presence of the *intonachino* containing a high percentage of binder in the samples of ancient Roman mortar (Campanella *et al.*, 1998b) [14], which is absent in the samples of Renaissance mortar (Campanella *et al.*, 1998a) [13]. The percentage of organic substance in the latter may be attributed to the relatively small but nevertheless more significant traces of more or less burnt straw contained in the Renaissance *arriccio* samples.

Lastly, also in this case it was demonstrated how, also in the present research, the coupling of thermal analysis and chemometrics represents a useful, simple and advantageous approach for dealing with problems of characterization and classification of different kinds of archaeological finds and cultural heritage.
